# A new species of *Peniculus* (Copepoda: Siphonostomatoida) parasitizing mesopelagic myctophid fish: first discovery of colonization of the genus in deep water

**DOI:** 10.1051/parasite/2018057

**Published:** 2018-11-26

**Authors:** Susumu Ohtsuka, Jun Nishikawa, Geoffrey A. Boxshall

**Affiliations:** 1 Takehara Station, Seotuchi Field Science Center, Graduate School of Biosphere Science, Hiroshima University 5-8-1 Minato-machi Takehara Hiroshima 725-0024 Japan; 2 Department of Marine Biology, School of Marine Science and Technology, Tokai University Orido, Shimizu Shizuoka 424-8610 Japan; 3 Life Sciences Department, The Natural History Museum Cromwell Road London SW7 5BD United Kingdom

**Keywords:** colonization, diel vertical migration, mesopelagic, Myctophidae, *Peniculus*, Pennellidae

## Abstract

*Peniculus hokutoae* n. sp. is described on the basis of an ovigerous adult female parasitizing the caudal fin of the myctophid fish *Symbolophorus evermanni* (Gilbert, 1905), collected from Suruga Bay, Japan. This is the first record of parasitism by this genus on mesopelagic myctophid fish. The new species is easily distinguished from other congeners in: (1) the presence of a conical process anterior to the rostrum; (2) the secondary elongation of the first pedigerous somite; (3) the incorporation of the third and fourth pedigerous somites into the trunk; (4) the unilobate maxillule bearing two unequal apical setae; (5) the lack of any processes on the first segment of the maxilla. Four morphological patterns of the cephalothorax, neck and anterior parts of the trunk can be found in the genus. We infer that initial colonization of a mesopelagic myctophid fish as host is likely to have occurred when the diurnally-migrating myctophid host was feeding in near-surface waters at night and was exposed to infective stages of *Peniculus*.

## Abstract (fr)

*Peniculus hokutoae* n. sp. est décrit sur la base d'une femelle adulte ovigère parasitant la nageoire caudale du poisson Myctophidae *Symbolophorus evermanni* (Gilbert, 1905), prélevée à Suruga Bay, au Japon. Il s'agit du premier signalement de parasitisme de ce genre chez les poissons myctophidés mésopélagiques. La nouvelle espèce se distingue facilement des autres congénères par : (1) la présence d’un processus conique antérieur au rostre ; (2) l'élongation secondaire du premier somite pédigère ; (3) l'incorporation des troisième et quatrième somites pédigères dans le tronc ; (4) le maxillule unilobé portant 2 soies apicales inégales ; (5) l'absence de processus sur le premier segment du maxillaire. Quatre types morphologiques du céphalothorax, du cou et des parties antérieures du tronc peuvent être trouvés dans le genre. Nous en déduisons que la colonisation initiale d'un poisson myctophidé mésopélagique en tant qu'hôte a probablement eu lieu lorsque l'hôte myctophidé, à migration diurne, se nourrissait la nuit dans des eaux proches de la surface et a été exposé aux stades infectants de *Peniculus*.

## Introduction

The siphonostomatoid family Pennellidae is a group of highly transformed copepods typically infesting fish as their final hosts, as meso- or ectoparasites [4]. The family currently accommodates 24 valid genera [4, 5, 20]. The genera *Peniculus* von Nordmann, 1832 and *Pennella* Oken, 1815 tend to parasitize shallow-water fish, while other genera, such as *Sarcotretes* Jungersen, 1911, *Lernaeenicus* Lesueur, 1824, *Protosarcotretes* Ohtsuka, Lindsay & Izawa, 2018, *Cardiodectes* Wilson, 1917. *Ophiolernaea* Shiino, 1958 and *Parina* Kazachenko & Avdeev, 1977 infest deep-sea fish [1–5, 20]. Myctophid fish are one of main host groups for these deep-sea pennellids [3, 20].

**Figure 1:**
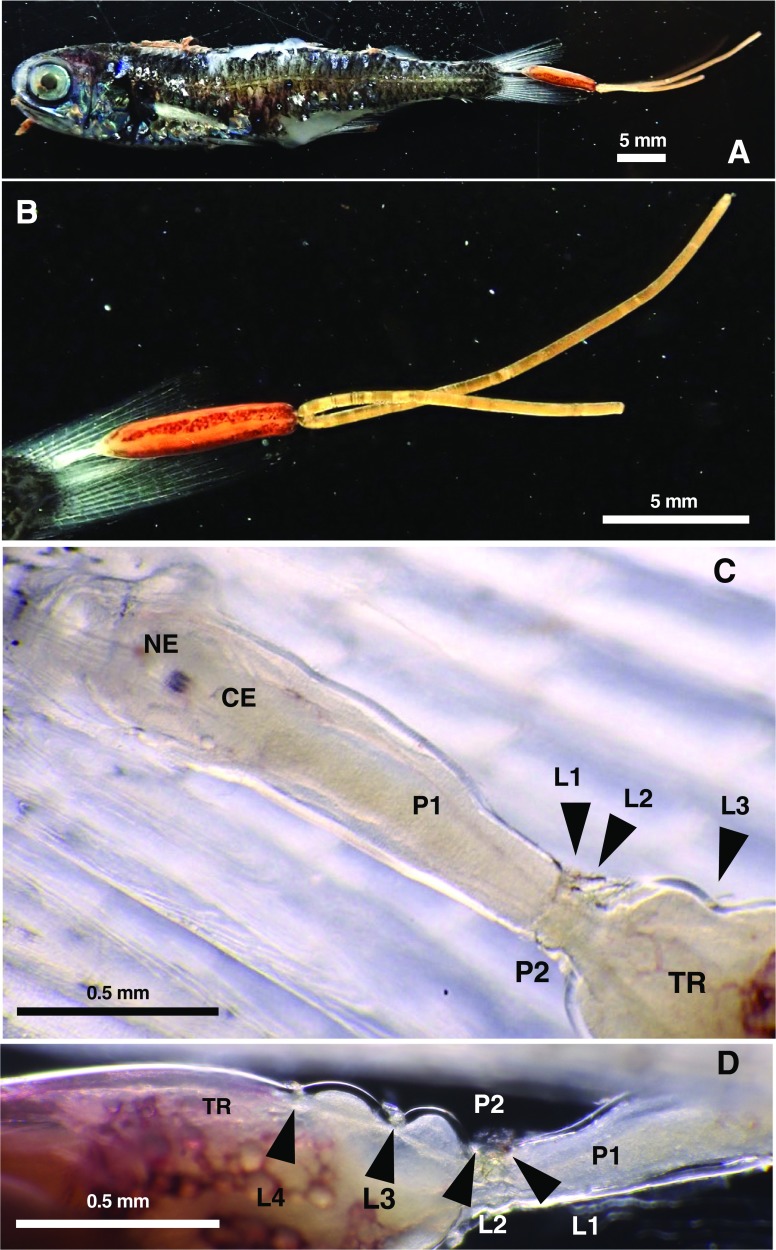
*Peniculus hokutoae* n. sp., holotype female. (A), (B) Living specimen *in-situ* on caudal fin of host *Symbolophorus evermanni* (Gilbert, 1905); (C) Cephalothorax and neck, dorsal view; (D) Neck and anterior part of trunk, lateral view. Abbreviations: CE: dorsal cephalic shield; L1–4: legs 1–4; NE: naupliar eye; P1, 2: pedigerous somites 1 and 2; TR: trunk.

During deep-sea zooplankton surveys in Suruga Bay, middle Japan by Tokai University, an undescribed species of the pennellid genus *Peniculus* infesting the caudal fin of a myctophid fish (Fig. 1A) was found, in addition to the recent discovery of a new pennellid genus and species *Protosarcotretes nishikawai* Ohtsuka, Lindsay & Izawa, 2018 [Japanese name: houraieso-no-namida (new), Japanese, meaning a tear drop of Pacific viper fish] parasitic on Pacific viper fish [20]. The present paper provides a taxonomic description of the undescribed new pennellid on the myctophid fish, together with notes on host-specificity, distribution and colonization of *Peniculus*.

## Materials and methods

A parasitic copepod specimen attached to the caudal fin of its host fish was collected in Suruga Bay (35˚03′20″ N, 138˚41′00″ E), Japan between 11:26 and 13:12 on July 12, 2018 in an oblique tow (0–917.9 m depth) of the ORI net (335 μm mesh, 1.6 m mouth diameter) [22] during cruise SRM-18-7-1 of the R/V Hokuto (Tokai University). The copepod specimen was photographed live before being preserved in 99.5% ethanol (see Figs. 1A and 1B). The only host fish in the plankton sample was identified as *Symbolophorus evermanni* (Gilbert, 1905) by reference to Nakabo [18]. The copepod was observed in lactophenol using a Humes & Gooding’s [11] slide, and illustrated with the aid of a drawing tube attached to an Olympus microscope. Copepod terminology follows Huys & Boxshall [12]. The type specimen of the parasitic copepod and the host fish are deposited at the National Museum of Nature and Science, Tsukuba, Japan (NSMT).

### Taxonomy

Order Siphonostomatoida Thorell, 1895
Family Pennellidae Burmeister, 1835
Genus *Peniculus* von Nordmann, 1832

*Remarks*. Castro-Romero [7] redefined the diagnosis of the genus *Peniculus* together with that of other pennellid genera *Metapeniculus* Castro-Romero & Baeza-Kuroki, 1985 and *Trifur* Wilson, 1917. However, some important characteristics were missing from the diagnosis of *Peniculus*: lack of antennules, for example. Although Boxshall & Halsey [4] regarded the incorporation of one or more of the posterior pedigerous somites into the trunk as a key character in defining *Peniculus*, it was not mentioned in Castro-Romero’s [7] diagnosis. After comparisons among related congeners (cf. Fig. 3), this relatively stable feature should be added to the generic diagnosis: pediger 4 or pedigers 3 and 4 incorporated into trunk.

The presence or absence of rami of legs is not mentioned in previous definitions of the genus, basically because the rami of the legs are typically missing in mature adult females. However, our observations of the new species described below have revealed remnants or scars of rami on the basis of the legs. This indicates that legs 1 and 2 are biramous, while legs 3 and 4 are uniramous. This configuration of the legs resembles that of other pennellid genera such as *Sarcotretes* and *Lernaeenicus* that retain the rami on the legs in the adult female (see Table 1 in [20]).

#### *Peniculus hokutoae* n. sp. [Japanese name: hokuto-kozutsu-hijikimushi (new)] (Figs. 1 and 2)

urn:lsid:zoobank.org:act:F0234087-38B2-4B36-9649-F7CBE10B1FFC

**Figure 2:**
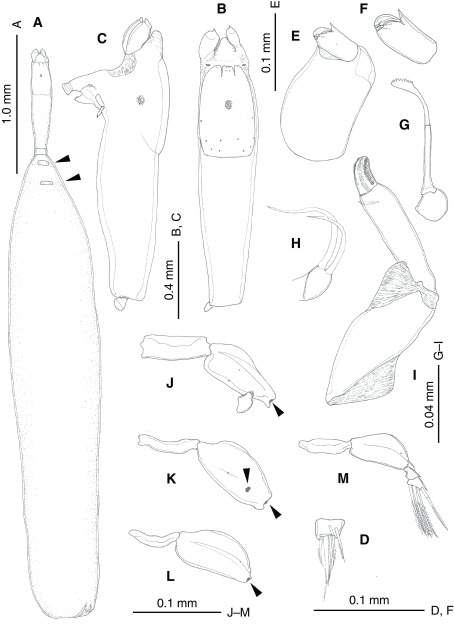
*Peniculus hokutoae* n. sp., holotype female. (A) Habitus, dorsal view, posterior part twisted, tergites on third and fourth pedigerous somites arrowed; (B) Cephalothorax, dorsal view; (C) Cephalothorax, lateral view; (D) Caudal ramus; (E) Antenna; (F) Terminal segment of antenna; (G) Mandible; (H) Maxillule; (I) Maxilla; (J) Leg 1, anterior view; (K) Leg 2, anterodistal view; (L) Leg 3, anterior view; (M) Leg 4, anterior view. Arrows in (J) to (L) indicate bases of rami.

*Type*. Holotype: ovigerous adult female attached to caudal fin of *Symbolophorus evermanni* (NSMT-P 132778), total length 50.9 mm, collected at depths of 0–917.9 m in Suruga Bay (35˚03′20″ N, 138˚41′00″ E), July 12, 2018; dissected, cephalothorax, mouthpart appendages and legs 1 and 2 in one slide, trunk and eggs in a vial (NSMT-Cr 25880).

*Type-locality:* Suruga Bay (35˚03′20″ N, 138˚41′00″ E), Japan.

*Description*. Ovigerous adult female. Body (Figs. 1A and 2A) comprising cephalothorax, comprising cephalosome covered with dorsal shield fused to secondarily elongate pediger 1, small pediger 2 and cylindrical trunk in which pedigers 3 and 4 completely incorporated. Total length from anterior tip of cephalosome to posterior end of trunk 7.95 mm. Cephalosome (Figs. 1C and 2A–2C) anteriorly produced into conical tip. Dorsal cephalic shield (Fig. 2B) trapezoidal, with anterior margin divided into three parts clearly visible in dorsal view. Middle part representing rostrum (Fig. 2B) truncate at tip, ornamented with two pairs of hair-sensilla. Proboscis moderately produced ventrally, carrying oral cone at tip. Buccal cone without observed slits or scales. Pediger 1 fused to cephalosome (Figs. 1A and 2A–2C) secondarily elongated, *ca.* 1.6 times as long as dorsal cephalic shield, tapering posteriorly. Pediger 2 (Fig. 2A) small, wider than long. Trunk (Fig. 2A) about 4.6 times as long as a combination of cephalosome and pedigers 1 and 2. Trunk about 5.2 times as long as wide. Pediger 3 separate from pediger 4. Tergites of pedigers 3 and 4 (Fig. 2A, arrowed) present. Abdomen vestigial. Paired gonopores located at posteriormost corner; two heavily chitinized ridges present medial to gonopore. Paired caudal rami (Fig. 2D) tiny, positioned close together subterminally; bearing six setae.

Antennule missing with paired scars visible (Fig. 2B). Antenna (Figs. 2E and 2F) heavily sclerotized, two-segmented, located on highly swollen, globular base; basal segment, expanded with two triangular processes; distal segment forming curved claw with sharply pointed tip, bearing minute setule basally. Mandible (Fig. 2G) slender, consisting of 3 parts: basal part globular; middle longest, cylindrical; distal recurved, with 10 teeth along inner margin of terminal expansion. Maxillule (Fig. 2H) unilobed with two unequal apical setae. Maxilla (Fig. 2I) two-segmented: basal segment robust, unarmed; distal segment with subterminal spinular row; terminal element small, ornamented with three rows of prominences.

Legs 1–4 all with partially divided protopod and intercoxal sclerite. Legs 1 (Fig. 2J) and 4 (Fig. 2M) each retaining remnant of rami. Legs 1 and 2 (Fig. 2K) biramous, while legs 3 (Fig. 2L) and 4 (Fig. 2M) uniramous, based on articulation scars on protopods. Left leg 4 (Fig. 2M) with two-segmented exopod, armed with outer seta on outer terminal corner of protopod; first segment unarmed, second segment with three setose setae, one weakly sclerotized seta and 1 minute spiniform seta. Right leg 4 lacking ramus.

Egg string linear (Figs. 1A and 1B): left egg string longer than body; right egg string incomplete.

*Coloration*. The specimen was still alive on collection, although the host was dead. The body coloration is based on this live specimen. The cephalothorax was translucent except for the naupliar eye (Figs. 1C and 1D). The trunk was entirely tinged with reddish brown with numerous pigmented droplets dorsally (Fig. 1B). Paired ovaries were yellowish in color (Fig. 1B). Eggs were light orange in color (Figs. 1A and 1B).

*Remarks*. The taxonomy of the genus *Peniculus* and related genera (*Metapeniculus*, *Propeniculus* Castro-Romero, 2014, *Pseudopeniculus* Castro-Romero, 2014) was revised by Castro-Romero [7] and Castro-Romero *et al.* [9]. The morphological features of the new species essentially fall within the diagnosis of *Peniculus* as redefined by Castro-Romero [7], although the structures of the maxillule, maxilla and legs differ slightly from those of other congeners.

**Figure 3:**
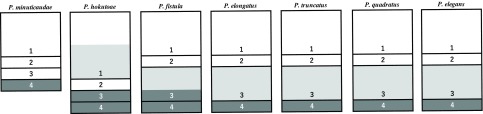
Schematic illustration of cephalothorax and pedigerous somites 1–4 of seven species of *Peniculus*. Numbers indicate relative positions of legs on pedigerous somites 1–4. Light gray: elongated part; dark gray: trunk.

The present new species is easily distinguished from other congeners by: (1) the secondary elongation of the first pedigerous somite (Fig. 3); (2) the third and fourth pedigerous somites are included in the trunk (Fig. 3); (3) the frontal expansion anterior to the rostrum; (4) unilobed maxillule bearing two terminal setae of unequal lengths; (5) the lack of a process on the first segment of the maxilla.

In the type specimen of the new species, some rami of legs still remain and the possibility of accidental detachment of the other rami during handling and observation of the specimen for this description cannot be ruled out.

*Etymology*. The new species is named after the research vessel Hokuto, of Tokai University.

## Discussion

### Taxonomy

The taxonomy of the genus *Peniculus* is still confused, although Castro-Romero [7], Castro-Romero and Kuroki [8], and Castro-Romero *et al.* [9] have revised the generic diagnosis. They tentatively recognized nine species in the genus: *P. calamus* von Nordmann, 1864; *P. clavatus* (Müller, 1799); *P. communis* Leigh-Sharpe, 1934; *P. elegans* Leigh-Sharpe, 1934; *P. elongatus* Boxshall, 1986; *P. fistula* von Nordmann, 1832; *P. minuticaudae* Shiino, 1956; *P. ostraciontis* Yamaguti, 1939; and *P. truncatus* Shiino, 1956. However, Kabata and Wilkes [14] pointed out that *P. calamus* and *P. fissipes* Wilson, 1917 can be relegated to synonymy with the type species *P. fistula*. Castro-Romero [7] included *P. calamus* in the genus without any comment on the proposal of Kabata and Wilkes [14].

Castro-Romero [7] established the two new genera *Propeniculus* and *Pseudopeniculus* to accommodate two species formerly placed in *Peniculus*. These type species are *Peniculus trichiuri* Gnanamuthu, 1951 (senior synonym of *P. theraponi* Gnanamuthu, 1951, *P. sciaenae* Gnanamuthu, 1951, *P. scomberi* Gnanamuthu, 1951, *P. stromatei* Gnanamuthu, 1951) and *Peniculus asinus* Kabata & Wilkes, 1977, respectively. In the same year, Moon and Choi [17] independently described a new species of *Peniculus, P. quadratus* Moon & Choi*,* 2014 from Korea, and mentioned that 15 species were accommodated in the genus, including species now belonging to *Propeniculus* and *Pseudopeniculus*. Currently [5], 10 species, *i.e.*, Castro-Romero’s [7] nine species and *P. quadratus* are listed as valid in the genus. However, the description of *P. communis* by Leigh-Sharpe [15] is inadequate by modern standards and the exact identity cannot now be confirmed. The absence of any sign of the buccal cone in *P. communis* indicates a possible similarity to *Propeniculus,* but in this species the protopods of legs 1–4 can be clearly seen in Leigh-Sharpe [15], while in *Propeniculus* no legs were observed. *Peniculus clavatus* was redescribed by Leigh-Sharpe and Perkins [16] and appears to differ rather remarkably from other congeners, in the presence of a special frontal organ and antennules (see Fig. 3 in [16]). After consideration of the position and shape of this “frontal organ”, we infer that this structure is probably the grasping antennae which were misinterpreted. The morphology of the antennae and maxillules also differs greatly from that of other congeners. Given these problems, we believe that these two species should be treated as *incertae sedis*. As pointed out by many taxonomists, a systematic revision of *Peniculus* based on modern taxonomic techniques is needed.

Based on the seven well-described species, four morphological patterns of the cephalothorax, neck and anterior parts of the trunk can be found (Fig. 3). In *P. minuticaudae*, the cephalothorax and neck are not elongate, and only the fourth pedigerous somite is incorporated into the trunk. In *P. hokutoae* n. sp., the part of the cephalothorax representing the first pedigerous somite is secondarily elongated, and both the third and fourth pedigerous somites are involved in the trunk. In the other five species, elongation of the third pedigerous somite is found. In *P. fistula*, the third and fourth pedigerous somites are both incorporated into the trunk, while in the other four species, only the fourth is involved.

### Host and distribution

The known hosts and geographical distributions of *Peniculus* species are summarized in Table 1. Surprisingly, *P. fistula* (including *P.* cf. *fistula* sensu Castro-Romero *et al.* [9]) infests a wide range of hosts (two superorders, six orders, 20 families, 42 species) occurring across the world. Castro-Romero *et al.* [9] reported low genetic diversity of the populations of *P*. cf. *fistula* parasitic on nine species of hosts occurring off the coast of Chile, irrespective of some intraspecific morphological variation. In contrast, other species exhibit relatively limited host-specificity in a restricted geographical area. For example, the hosts of *P. minuticaudae* and *P. truncatus* seem to be restricted to East Asian fish belonging to the Monacanthidae and Sebastidae, respectively. One exceptional case in *P. minuticaudae* was parasitism on brown-banded butterflyfish *Rao modesta*, but this seems to have occurred in the artificial conditions of an aquarium tank [21].

**Table 1:** Host, attachment site and distribution of *Peniculus*. –: no data.

Species	Host (attachment site)	Distribution	Reference
*P. calamus* von Nordmann, 1864	unknown	Hawaii	[25]
*P. clavatus* (Müller, 1799)	*Sebastes norvegicus* (Ascanius) (fin)	Iceland	[16]
	*S. norvegicus* (fin), *S. mentella* Travin (fin)	Eastern North Atlantic	[28]
*P. communis* Leigh-Sharpe, 1934	*Atherina forskali* Rupp (fin), *Atherina pinguis* Lac (tail)	Ambon	[15]
*P. elegans* Leigh-Sharpe, 1934	*Chromis caerulea* (Cuvier) (as *C. lepidurus*) (–)	Anchorage Salomakie Is.	[15]
*P. elongatus* Boxshall, 1986	*Pempheris affinis* McCulloch (–)	New South Wales, Australia	[1]
*P. fistula* von Nordmann, 1832*	*Chromis notata* (Temminck & Schlegel) (fin, body surface)	Chuja Island, Korea	[17]
	*Anisotremus scapularis* Tschudi (fin)	South Pacific coast of Chile	[9]**
	*Cheilotrema fasciatum* Tschudi (fin)		
	*Chromis crusma* Valenciennes (fin)		
	*Girella laevifrons* Tschudi (fin)		
	*Hemilutjanus macrophthalmus* Tschudi (fin)		
	*Isacia conceptionis* Cuvier (fin)		
	*Mugil cephalus* Linnaeus (fin)		
	*Odontesthes regia* Humboldt (fin)		
	*Prolatilus jugularis* Valenciennes (fin)		
*P. hokutoae* n. sp.	*Symbolophorus evermanni* (Gilbert) (fin)	Suruga Bay, Japan	Present study
*P. minuticaudae* Shiino, 1956	*Stephanolepis cirrhifer* (Temminck & Schlegel) (fin)	Shirahama, Wakayama, Japan	[23]
	*Stephanolepis cirrhifer* (fin)	Oita, Japan	[19]
	*Thamnaconus modestus* (Gunther) (fin)	Oita, Japan	[19]
	*Thamnaconus modestu*s (fin)	Tongyeong, Korea	[24]
	*Aluterus monoceros* (Linnaeus) (fin)	Aquarium in Kagoshima, Japan	[21]
	*Paramonacanthus japonicus* (Tileslus) (fin)	Uwajima, Ehime, Japan	[13]
	*Roa modesta* (Temminck & Schlegel) (fin)		
	*Stephanolepis cirrhifer* (fin)		
*P. ostraciontis* Yamaguti, 1939	*Tetrosomus gibbous* Linnaeus (head)	Pacific	[29]
	*Tetrosomus concatenatus* (Bloch) (head)	Sagami Bay, Japan	[23]
*P. quadratus* Moon & Choi, 2014	*Neoditrema ransonnetii* Steindachner (mouth palate)	Chuja Island, Korea	[17]
*P. truncatus* Shiino, 1956	*Sebastes oblongus* (Gunther) (fin)	Off Wagu, Mie, Japan	[23]
	*Sebastes schlegelii* Hilgendorf (fin)	Kamak Bay, Korea	[10]
	*Sebastes schlegelii* (fin)	Tongyeong, Korea	[24]

* As for *P. fistula,* hosts are listed on the basis of two references published after Bunkley-Williams & William [6] in which two superorders, six orders, 19 families and 33 species of host fish were recorded worldwide.

** Castro-Romero *et al.* [9] identified the taxon examined as *Peniculus* cf. *fistula*.

It is most likely that the host-usage of *Peniculus* species other than *P. hokutoae* n. sp. is restricted to coastal or epipelagic fish. In contrast, the host of *P. hokutoae* n. sp. is a mesopelagic myctophid *Symbolophorus evermanni* [18]. This is the first record of the occurrence of any *Peniculus* on a member of the family Myctophidae. This host family is frequently utilized by deep-sea pennellids including species of *Sarcotretes*, *Lernaeenicus* and *Cardiodectes* [20], but never before by a *Peniculus*. This parasitism could be explained by the following evolutionary scenario. Myctophids are mainly distributed in meso- and bathypelagic zones, and many species exhibit diel vertical migration [18, 26, 27]. *Symbolophorus evermanni* is one of the dominant myctophid fish that occur nocturnally in surface waters of the Kuroshio region in the northwestern Pacific (Watanabe and Kawaguchi [26]). The myctophid is distributed at depths of 600–1150 m during daytime and upper 125 m at night [18, 26, 27] and can be ecologically classified as a migrant [27]. Exposure to infective stages of the *Peniculus* could have occurred nocturnally while the hosts were in the surface waters. Once on the new host, the copepod would eventually have adapted to its deep-sea myctophid host.
